# Cryptocephal, the *Drosophila melanogaster* ATF4, Is a Specific Coactivator for Ecdysone Receptor Isoform B2

**DOI:** 10.1371/journal.pgen.1002883

**Published:** 2012-08-09

**Authors:** Sebastien A. Gauthier, Eric VanHaaften, Lucy Cherbas, Peter Cherbas, Randall S. Hewes

**Affiliations:** 1 Department of Biology, University of Oklahoma, Norman, Oklahoma, United States of America; 2 Department of Biology, Indiana University, Bloomington, Indiana, United States of America; University of California San Francisco, United States of America

## Abstract

The ecdysone receptor is a heterodimer of two nuclear receptors, the Ecdysone receptor (EcR) and Ultraspiracle (USP). In *Drosophila melanogaster*, three EcR isoforms share common DNA and ligand-binding domains, but these proteins differ in their most N-terminal regions and, consequently, in the activation domains (AF1s) contained therein. The transcriptional coactivators for these domains, which impart unique transcriptional regulatory properties to the EcR isoforms, are unknown. Activating transcription factor 4 (ATF4) is a basic-leucine zipper transcription factor that plays a central role in the stress response of mammals. Here we show that Cryptocephal (CRC), the *Drosophila* homolog of ATF4, is an ecdysone receptor coactivator that is specific for isoform B2. CRC interacts with EcR-B2 to promote ecdysone-dependent expression of ecdysis-triggering hormone (ETH), an essential regulator of insect molting behavior. We propose that this interaction explains some of the differences in transcriptional properties that are displayed by the EcR isoforms, and similar interactions may underlie the differential activities of other nuclear receptors with distinct AF1-coactivators.

## Author Summary

Nuclear receptors are proteins that regulate gene expression in response to steroid and thyroid hormones and other small lipid-soluble signaling molecules. In many cases, nuclear receptor genes encode multiple variants (isoforms) that direct tissue- and stage-specific hormonal responses. The sequence differences among isoforms are often found at the protein N-terminus, which mediates hormone-independent interactions with unknown regulatory partners to control target gene expression. Here, we show that the fruit fly Cryptocephal (CRC) protein is a specific coactivator for one of three isoforms of the receptor for the insect molting steroid, ecdysone. Our findings reveal a mechanism for differential activation of gene expression in response to ecdysone during insect molting and metamorphosis, and contribute to our understanding of isoform-specific functions of nuclear hormone receptors.

## Introduction

Nuclear receptors are multifunctional transcription factors that mediate responses to steroids and other small hydrophobic signaling molecules. Most nuclear receptors have two transcriptional activation functions (AF1 and AF2). AF2 is formed by ligand-induced folding of the ligand-binding domain, and the structural basis of its interaction with coactivators is becoming known. AF1 designates a second, ligand-independent activation function often present in the N-terminal region of the receptor. AF1 sequences are not conserved, and the existence of an AF1 must be inferred from functional assays. Although the AF1s are of considerable interest, because they often differentiate receptor isoforms and because some have been shown to interact with general transcription factors, comparatively few AF1-coactivator interactions have been characterized. The relative contributions of AF1 and AF2 to transcriptional activation vary among receptors, and for any given receptor the relative contributions may depend upon the promoter context [1].

The three isoforms of EcR (FlyBase ID: FBgn0000546) have unrelated AF1 regions, each capable of mediating transcriptional activation in some contexts [2]–[6]. Although several coactivators and corepressors for the AF2 of EcR have been identified [7]–[13], the interacting factors for the unique AF1 domains remain unknown. The 17-residue AF1 region of isoform B2 is capable of strong transcriptional activation on a standard test promoter and is required for ecdysone-regulated differentiation in a few fly tissues [2], [4]. Here, we show that the bZIP transcription factor, CRC (FBgn0000370), binds the AF1 of isoform B2 to promote steroid-dependent expression of the peptide molting hormone, ETH (FBgn0028738; [14]).

## Results

### The AF1 of EcR-B2 Bound to the Leucine Zipper of CRC

**Figure 1:**
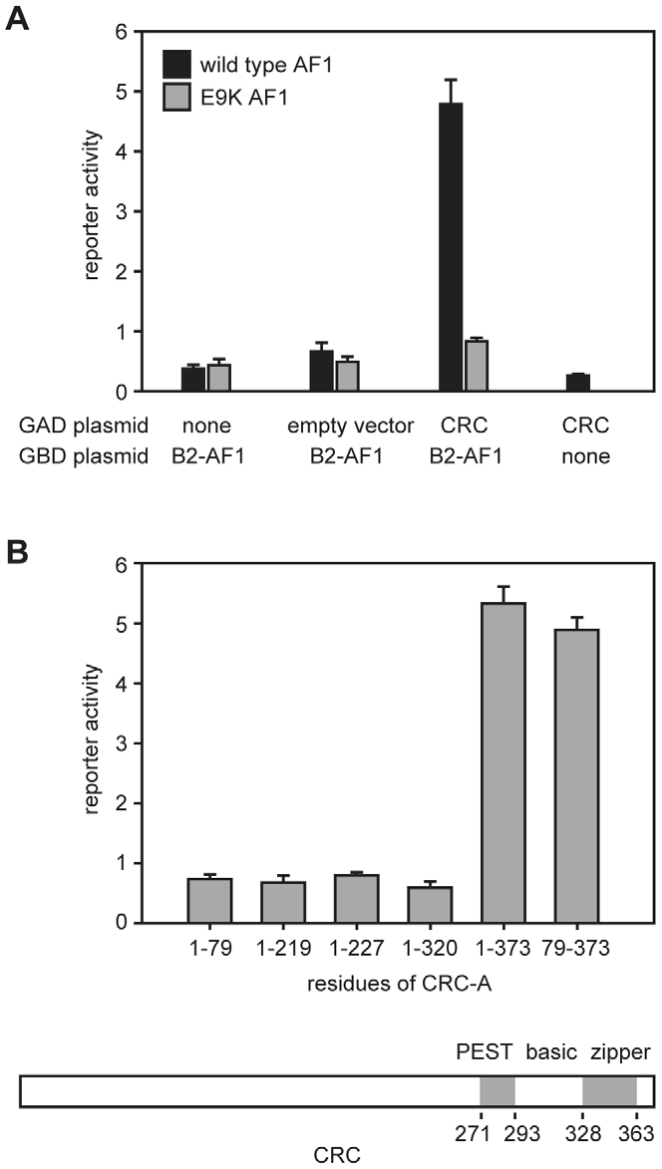
Interaction of the EcR-B2 N-terminus with CRC in yeast two-hybrid assay. (A) In yeast two-hybrid assays, activation of a *UAS-lacZ* reporter (reporter activity) required the presence of both EcR-B2 and CRC-A. This interaction was abolished by the EcR-B2 mutation E9K. In these assays, a fusion of the GAL4 DNA-binding domain to either the 17-residue AF1 domain of EcR-B2 (black bars), or the same fragment containing the mutation E9K (gray bars) served as bait. Full length CRC-A was used as the prey. Error bars indicate standard deviations for 4 independent assays. (B) The indicated CRC residues were substituted for full-length CRC-A and tested for binding to the wild-type EcR-B2 fusion fragment in yeast two-hybrid assays. Coordinates of the conserved domains of CRC are indicated in the drawing below.

We performed a yeast two-hybrid screen using the N-terminal region of EcR-B2 as bait and recovered a plasmid containing the complete coding sequence of the predominant CRC isoform, CRC-A [15]. Figure 1 illustrates the salient features of these assays. Interaction (as judged by reporter activation) required the presence of both CRC-A and the AF1 of EcR-B2 (Figure 1A). The EcR-B2 mutation E9K, which sharply reduces transcriptional activation *in vivo* [4], also abolished the two-hybrid interaction (Figure 1A). The interaction surface provided by CRC was contained within the C-terminal three-quarters of the protein, a region that includes its bZIP and PEST domains, and C-terminal truncation of the protein to remove just the leucine zipper domain abolished the interaction with EcR-B2 (Figure 1B).

**Figure 2:**
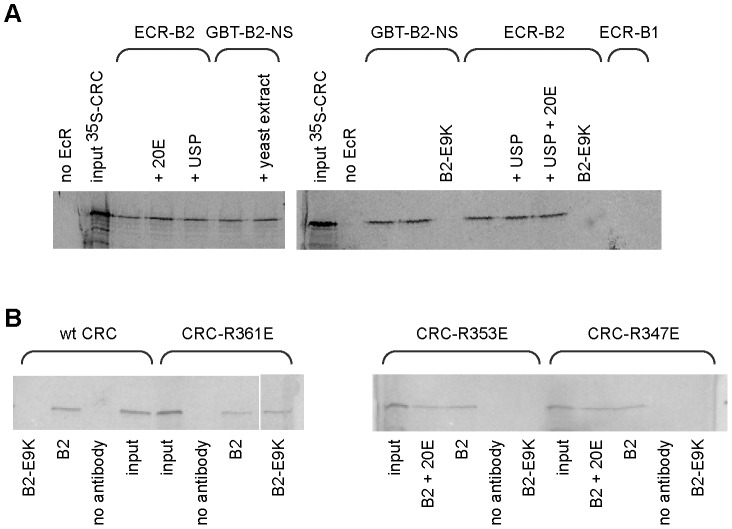
Binding of CRC to EcR-B2 *in vitro*. (A) CRC-A bound in vitro to the 17 amino acid amino-terminus of EcR-B2 and full length EcR-B2, but not to EcR-B1 or EcR-B2-E9K. This interaction was not dependent upon USP or ecdysone. Gels from two experiments are shown; the labeled protein was ^35^S-GAD-CRC-A in the left gel, ^35^S-CRC-A in the right gel. Lanes labeled “input” contained 100% of the input labeled protein. The remaining samples contained the EcR or EcR fragment shown above, and other components as indicated. GBT-B2-NS is a fusion of the DNA-binding domain of GAL4 with the amino-terminal 17 amino acids of EcR-B2. In lanes labeled B2-E9K, E9K mutant EcR-B2 or E9K mutant GBT-B2-NS was substituted 1∶1 for the corresponding wild-type protein. Full-length EcRs were precipitated with a mixture of two EcR-common region monoclonal antibodies, and GBT-B2-NS was precipitated with an antibody to the GAL4 DNA-binding domain. (B) A similar experiment, showing the binding of wild-type and mutant CRCs to full-length EcR-B2. Each incubation mixture contained the ^35^S-radiolabeled CRC protein listed above and the other components listed below, and precipitations were performed with the same mix of EcR common region antibodies as in (A). Wild-type CRC, CRC-R347E, and CRC-R353E bound to EcR-B2 but not EcR-B2-E9K. However, the basic-to-acidic mutation in CRC-R361E permitted binding to EcR-B2-E9K.

Binding of CRC to the amino-terminal region of EcR-B2 was confirmed *in vitro* (Figure 2A). Radiolabeled CRC-A bound to the EcR-B2 amino-terminus and to full-length EcR-B2, but not to EcR-B1 or to EcR-B2 carrying the E9K mutation. The AF1 region of the EcR-B2 N-terminus contains sequences suggestive of a short amphipathic helix, and it is known that the acidic-to-basic substitution E9K (within the proposed helix) abolishes AF1 function *in vivo* [4]. We think it likely that this helix is unstructured in solution and infer that both ionic and hydrophobic interactions play roles in its dimerization, probably with the leucine zipper region of CRC. To test this idea further, we made several individual basic-to-acidic mutations within the CRC leucine zipper – at sites predicted to determine the dimerization specificity of the bZIP domain [16] – and tested the binding of the mutant CRC proteins to wild-type and E9K mutant EcR-B2 (Figure 2B). The binding properties of CRC-R347E and CRC-R353E were indistinguishable from those of wild-type CRC, but CRC-R361E bound EcR-B2-E9K. That an alteration in CRC reversed the effect of an EcR mutation strongly implies direct interaction.

Neither USP (FBgn0003964) nor the hormone ecdysone affected the CRC-EcR-B2 interaction as measured in our biochemical tests (Figure 2A). While AF1 activity is hormone-dependent *in vivo*, that is probably due to the effects of corepressors (*e.g.* SMRTER) that bind unliganded EcR/USP and suppress the activity of AF1 [4].

### Loss of CRC Enhanced Phenotypes in Tissues Requiring EcR-B2

Both the *crc* mutant phenotype, which includes molting defects that result in supernumerary mouthparts in larvae and failure to evert the adult head at pupal ecdysis, and the pattern of *crc* expression suggest a role for CRC in the ecdysone response [15]. We used a genetic interaction test to determine whether CRC functions as a modulator of EcR-B2 function in flies. We examined the effects of a single copy of *crc^1^* (a spontaneous mutation, Q171R; FBal0001818) [15], [17] on the phenotype produced by targeted expression of the dominant-negative mutant EcR-B1-F645A [2], [4]. EcR-B1-F645A is normal in transcriptional repression (the effect of unliganded receptor), but it fails to mediate transcriptional activation. Because the EcR isoforms do not display isoform specificity in DNA binding [5], the EcR-B1-F645A mutant is thought to competitively inhibit all three endogenous EcR isoforms [18]. Hence, a reduction in EcR-B2 coactivator titer should selectively enhance the effects caused by EcR-F645A expression only in tissues requiring the EcR-B2 isoform.

Targeted expression of the dominant-negative receptor EcR-B1-F645A permits an examination of the properties of EcR function in specific tissues in the context of an otherwise normal animal [2]. *crc^1^* is a recessive mutation, and *crc^1^/crc^+^* heterozygous cells are phenotypically normal. We generated sensitized tissues by targeting expression of EcR-B1-F645A to five different developmental domains, using specific GAL4 drivers [2]. As shown in Table 1, *crc^1^* was a dominant enhancer of the EcR dominant negative phenotype in the Eip domain (largely larval epidermis) and in the slbo domain (specialized portions of the follicular epithelium of the egg chamber), but it had no significant effect in the GMR domain (primarily retinal epithelium), the dpp domain (primarily A/P disc boundaries), or the Lsp2 domain (fat body). We have previously described the EcR isoform requirements in each of these domains [2]. There was a remarkable correlation: Where EcR-B2 is required for development, wild-type *crc* function was also required, and where normal development does not require EcR-B2, reduction of the CRC titer had little or no effect.

**Table 1:** Dominant effects of *crc^1^* on the phenotypes of EcR-B1-F645A expression.

	% Survival				
Driver (temperature)	*crc^+^*/*crc^+^*	*crc^1^*/*crc^+^*	Ratio heterozygote/wild-type	Effect of *crc^1^* on EcR-F645A phenotype	EcR isoform requirement
GMR (20°)	4.7 (21)	5.5 (40)	1.2	none	any
dpp (20°)	4.2 (33)	8.5 (72)	2.0	slight suppression	any
Lsp2 (25°)	3.7 (12)	9.5 (25)	2.6	slight suppression	any
Eip (16°)	5.1 (15)	1.0 (13)	0.27	enhancement	B2
slbo (25°)	qualitative assay			enhancement	B2

All flies contained, in addition to the indicated *crc* genotype, one copy of *UAS-EcR-B1-F645A* and one copy of the indicated driver. Quantitative data represent % viability to adult eclosion for the indicated genotype; the nature of the lethality in each case is described elsewhere [5]. Effects of EcR-B1-F645A in the slbo domain were assessed qualitatively by observing the tendency of eggs laid by an affected female to collapse. Each datum was produced by crossing a driver stock to either *UAS-EcR-B1-F645A/CyO* (*crc^+^*/*crc^+^*) or *UAS-EcR-B1-F645A crc^1^/CyO* (*crc^1^*/*crc^+^*). Survival was determined by comparing the number of adults recovered which carry the *UAS-EcR-B1-F645A*-containing chromosome with the number of adults carrying the *CyO* balancer; the number in parentheses indicates the number of EcR-B1-F645A-expressing adults recovered. In a control experiment to determine the relative strength of transgene expression in each domain, the Gal4 drivers were crossed to *UAS-nuclear-GFP* (FBti0012492), the tissues were fixed in 4% paraformaldehyde and washed, and the intensity of direct GFP fluorescence in single confocal sections (signal - background) was quantified. The mean intensities of GFP fluorescence were: dpp (FBti0002123) 71.6+/−9.6, GMR (FBti0002994) 59.3+/−6.3, slbo (FBti0023075) 50.9+/−9.4, Eip (FBtp0016770) 30.6+/−4.3, Lsp2 (FBti0018531) 25.8+/−4.8 (+/− SEM, n = 6). Thus, the intensity of driver expression was not correlated with the ability to enhance the EcR dominant negative phenotype.

### CRC Regulation of ETH Expression

In homozygous or hemizygous *crc^1^* mutant larvae, expression of ETH is markedly reduced [19]. The loss of *crc* has similar effects on expression of an *ETH-EGFP* reporter gene, which contains 382 bp of the *ETH* promoter and precisely recapitulates the native pattern of ETH expression [14], [19]. Thus, CRC up-regulates ETH expression.

**Figure 3:**
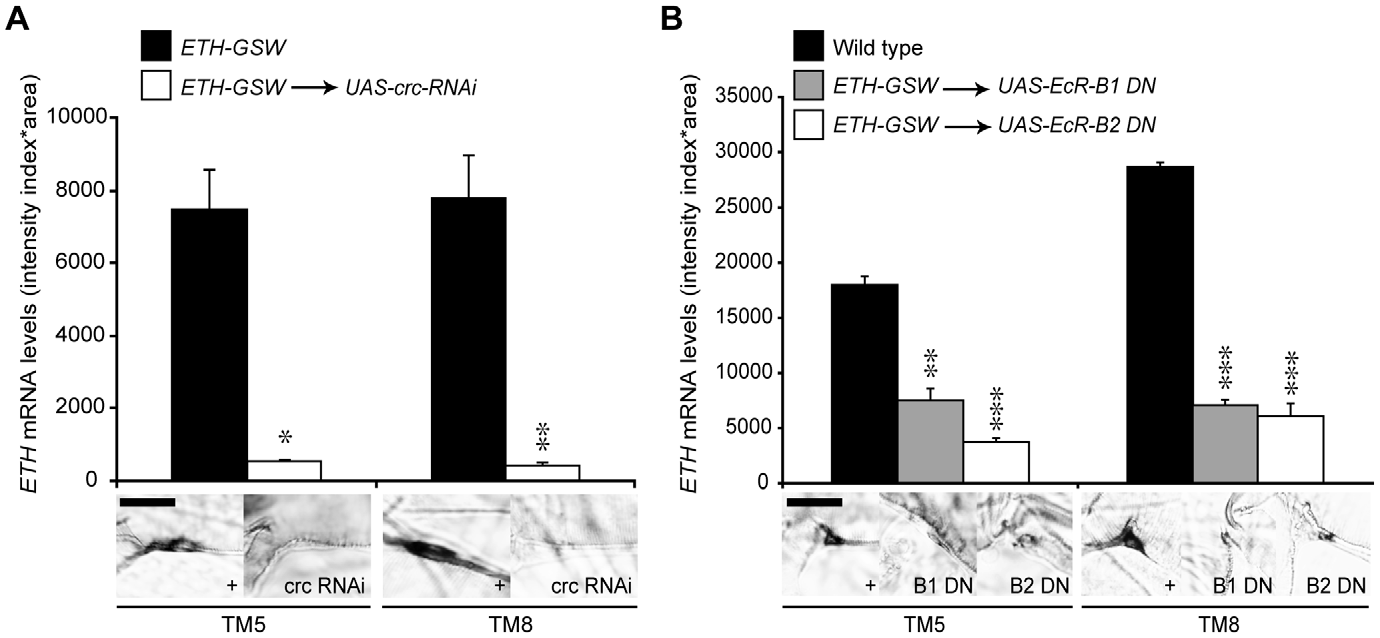
EcR-B1, EcR-B2, and CRC regulated *ETH* expression. (A) Inka cell-specific *crc* RNAi lowered *ETH* transcript levels (n = 9, *crc* RNAi; n = 6, *ETH-GSW*>+controls). *, p<0.05; **, p<0.01 (One-way ANOVA: TM5, p = 0.01225; TM8, p = 0.001375). (B) Inka cell-specific expression of dominant negative EcR-B1-W650A or EcR-B2-W650A (EcR-B1 DN or EcR-B2 DN) lowered *ETH* transcript levels (n = 9, dominant negatives; n = 23, Oregon R controls). **, p<0.01; ***, p<0.001 [One-way ANOVA with Bonferroni (all-pairwise) multiple comparison test: TM5, p = 0.000265; TM8, p = 0.000044]. Bar = 20 µM.

CRC is expressed in many larval tissues, including the endocrine source of ecdysone [15]. Therefore, to test for cell-autonomous regulation of *ETH* expression by CRC, we used an *ETH-GeneSwitch* driver to drive transgenic *crc* RNAi (*UAS-crc-RNAi*) specifically in the Inka cells (Figure S1), the site of ETH synthesis [14]. GeneSwitch is a conditional GAL4 protein that is activated by addition of the progesterone antagonist RU486 to the food [20]. Compared to the control larvae, larvae with *crc* RNAi showed a 15-fold or greater reduction in *ETH* transcript levels (Figure 3A). Thus, CRC was cell-autonomously required in the Inka cells for full *ETH* expression.

### Regulation of ETH Expression by Ecdysone

**Figure 4:**
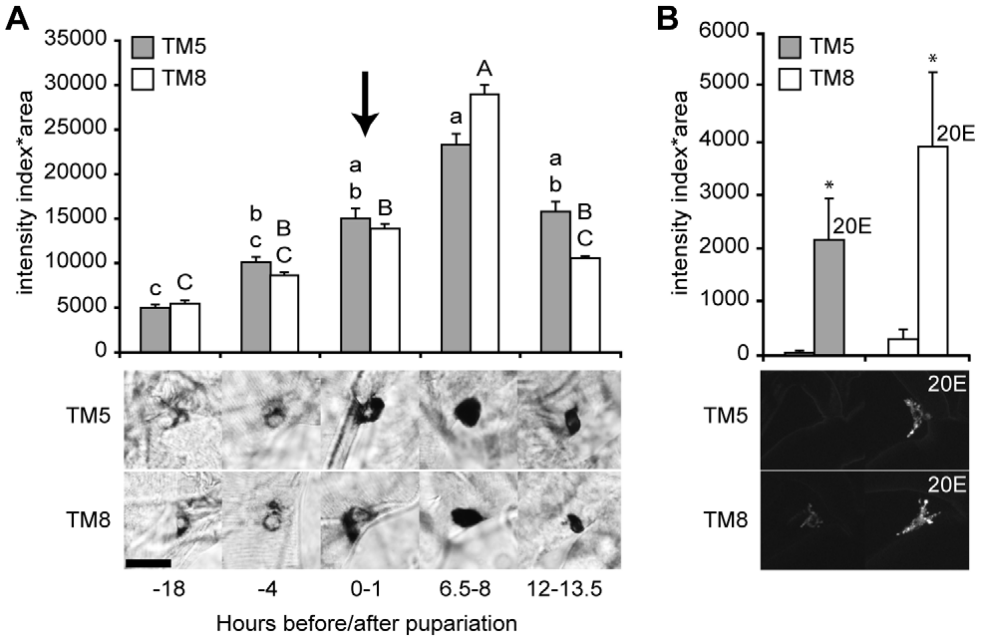
*ETH* expression was stimulated by ecdysone. (A) *ETH* transcript levels mirrored the changes in the circulating ecdysone titer with a 6.5 to 8 hr delay. Transcript levels were measured semi-quantitatively in as the intensity of *ETH in situ* hybridization in Inka cells in tracheal metamere 5 (TM5) and TM8 at five times at the onset of metamorphosis (relative to pupariation). The arrow indicates the peak of the late-larval pulse of circulating 20E, which occurs at pupariation [23]. Means with the same lower case (TM5) or upper case (TM8) letters were not significantly different [p>0.05, n = 5–8; One-way ANOVA (TM5, p = 0.000776; TM8, p<0.000001) with Bonferroni (all-pairwise) multiple comparison test]. Insets below the histograms in (A) and (B) show representative images of cells at each time point. (B) The Inka cells of young third instar larvae fed with 20E for 12 hr showed higher expression levels for the fluorescent ETH reporter, *ETH-EGFP* (n = 4). *, p<0.05 (one-way ANOVA: TM5, p = 0.036; TM8, p = 0.044). Bar = 20 µM.

The *Drosophila melanogaster ETH* promoter contains a putative ecdysone response element [19], [21], and ETH expression in the tobacco hawkmoth (*Manduca sexta*) fluctuates during the molts and is elevated in response to circulating ecdysteroids [22]. Therefore, we examined whether ETH expression is ecdysone-dependent. In larval and pupal Drosophila, expression of the ETH peptide hormone is restricted to 14 endocrine Inka cells located on the trachea [14]. We performed *ETH in situ* hybridization and found that *ETH* transcript levels increased gradually during the first few hours after metamorphosis was initiated (Figure 4A). The *ETH* transcript levels peaked 6–8 hr after the pulse of ecdysone that occurs at pupariation (Figure 4A), suggesting that *ETH* was transcribed in response to elevation of the circulating ecdysone titer.

We tested for direct ecdysone-dependence of ETH expression in young third instar stage larvae, when circulating ecdysone and *ETH* transcript and protein levels are low, by feeding them the major active form of ecdysone, 20-hydroxyecdysone (20E) [23]. These larvae carried the *ETH-EGFP* reporter gene. By 12 hr after the onset of the 20E treatment, the level of ETH-EGFP fluorescence was markedly elevated (Figure 4B). Thus, ETH expression was strongly up-regulated by circulating ecdysone.

To test for a direct, cell autonomous effect of ecdysone on ETH expression, we targeted EcR-F645A dominant negative proteins specifically to the Inka cells with the *ETH-GeneSwitch* driver. Following Inka cell expression of the EcR dominant negative proteins, *ETH* transcripts were still present but at levels that were 2–6 fold lower than in wild-type larvae (Figure 3B). Thus, ETH expression was strongly stimulated by ecdysone and required EcR expression in the Inka cells.

### CRC and EcR-B2 Interacted to Boost ETH Expression *In Vivo*

The EcR-B1-F645A mutant is effective as a dominant negative when it is expressed in excess of the wild-type isoforms (Table 1) [2], [4]. However, the dominant negative EcR-B1-F645A protein competes poorly with wild-type EcR when both are expressed from identical promoters [2], [4]. Therefore, to determine which EcR isoforms support up-regulation of *ETH* expression in the Inka cells, we performed competition experiments in which EcR-B1-F645A and individual wild-type isoforms were coexpressed under the control of the *ETH-GeneSwitch* driver. The ability of a wild-type EcR isoform to mitigate the effects of the dominant negative is indirect evidence in support of transcriptional activation of the *ETH* promoter by that isoform.

**Figure 5:**
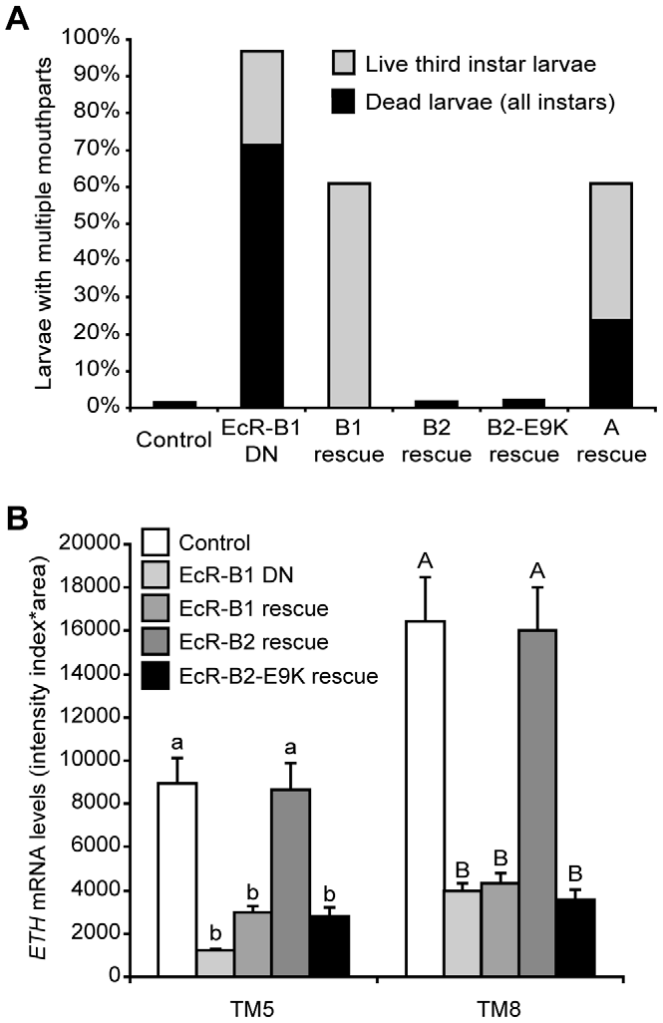
EcR-B2 rescued ecdysis and *ETH* expression following targeted expression of dominant negative EcR. (A) Ecdysis in third instar larvae expressing dominant negative EcR-B1-F645A only in the Inka cells (n = 98; EcR-B1 DN) was rescued by expression of EcR-B2 (n = 165) or EcR-B2-E9K (n = 143), but not by expression of EcR-B1 (n = 91) or EcR-A (n = 59). The controls had *ETH-GSW* only (n = 148), and all larvae were fed RU486. The larval counts included all live third instar larvae (with or without multiple mouthparts), as well as all dead larvae (of all stages). All of the dead larvae found had multiple mouthparts. (B) *ETH* expression in Inka cells expressing dominant negative EcR-B1-F645A isoform (n = 11) was rescued by co-expression of wild-type EcR-B2 (n = 8) but not wild-type EcR-B1 (n = 9) or the E9K mutant of EcR-B2 (n = 8). The controls had *ETH-GSW* only (n = 8), and all larvae were fed RU486. All larvae were dissected at ∼12 hr after ecdysis to the third instar. Means with the same lower case (TM5) or upper case (TM8) letters were not significantly different [p>0.05, One-way ANOVA (TM5, p<0.000001; TM8, p = 0.000002) with Bonferroni (all-pairwise) multiple comparison test].

Supernumerary mouthparts result when larvae fail to complete ecdysis to either the second or the third larval instar, and they are a characteristic feature of the *ETH* and *crc* mutant phenotypes [14], [15]. Over 95% of larvae with Inka cell-targeted EcR-B1-F645A expression had multiple mouthparts, and ∼70% of these animals died as larvae (Figure 5A). Simultaneous expression of wild-type EcR-B2 or EcR-B2-E9K with EcR-B1-F645A fully rescued lethality and ecdysis of the larval mouthparts, whereas EcR-B1 and EcR-A produced only partial rescue (Figure 5A). Within the Inka cells, *ETH* transcript levels were fully rescued by EcR-B2, but EcR-B1 and EcR-B2-E9K were ineffective at rescue (Figure 5B). Thus, of the three EcR isoforms, only wild-type EcR-B2 was capable of supporting full *ETH* expression and successful ecdysis. The E9K mutant of EcR-B2 failed to rescue *ETH* transcript levels, suggesting a model in which dimerization of EcR-B2 with CRC is required for *ETH* mRNA expression.

In *M. sexta*, 20E regulates ETH synthesis as well as the competency of the Inka cells to secrete ETH [24]. The ability of EcR-B2-E9K, and to a lesser extent EcR-B1 and EcR-A, to rescue ecdysis and lethality (Figure 5A) indicates that EcR likely regulates other Inka cell processes, such as ETH protein accumulation or secretory competence, that are necessary for signaling by ETH. We tested this hypothesis by performing ETH immunostaining in EcR-B2-E9K rescue animals both before and after secretion at pupal ecdysis. Although EcR-B2-E9K did not stimulate *ETH* transcription (Figure 5B), it drove ETH protein accumulation in the Inka cells (Figure S2A). Consistent with the predicted role of EcR in the development of secretory competence, we also observed a marked decrease in accumulated ETH at pupal ecdysis (Figure S2B). These results show that EcR—likely through different sets of EcR isoforms and transcriptional coactivators—regulates ETH protein accumulation independently of *ETH* mRNA expression.

## Discussion

Our experiments suggest that the 17-residue B2-specific N-terminus binds to the bZIP region of CRC, that an ionic interaction between EcR-B2-E9 and CRC-R361 plays some role in the binding, and that the interaction of the two proteins plays a crucial role in those tissues where EcR-B2 is essential. These tissues include the endocrine Inka cells, which display ecdysone-dependent upregulation of *ETH* transcripts and which require EcR-B2 and CRC for full *ETH* expression. Taken together, these findings implicate CRC as an isoform-specific transcriptional activator for EcR-B2.

In diverse systems, bZIP proteins interact with dyadic or palindromic promoter sequences as homodimers or heterodimers with other bZIP partners [25]. Dimerization involves regularly spaced hydrophobic amino acids that form a coiled-coil between two leucine zipper domains [26]. Other bZIP transcription factors are known to interact with nuclear receptors, modulating the activities of either AF1 or AF2 [27]–[29], but in the cases reported previously, bZIP proteins bind either to the DNA-binding domain or to the hinge domain of the nuclear receptor. By contrast, CRC (through its bZIP domain) appears to bind directly to the EcR-B2 AF1 region, and its interaction is specific to one EcR isoform.

ATF4, the mammalian homolog of CRC, plays a central role in stress responses [30]. The role of CRC in ecdysone signaling suggests the possibility of interesting and unexpected connections between stress responses and the control of developmental timing and metamorphosis.

The *ETH* promoter contains sequences matching the consensus half-sites for binding of ATF4 and EcR to DNA. These half-sites are separated by 4 nucleotides, and they are located within a highly conserved sequence (comparing *D. melanogaster* to several other *Drosophila* species) that is 138–171 nucleotides upstream of the *ETH* transcriptional start site [19]. Since bZIP proteins may bind first sequentially as monomers and then dimerize while bound to DNA [26], [31], these observations suggest a model in which CRC participates in the stabilization of EcR-B2 binding to the *ETH* promoter. This interaction provides a basis for understanding some of the differences in transcriptional properties that are displayed by the EcR isoforms and perhaps other nuclear receptors with distinct AF1-coactivators.

## Materials and Methods

### Yeast Two-Hybrid Screening

Yeast two-hybrid assays were carried out using the Clontech Matchmaker yeast two-hybrid kit (Clontech, Mountain View, CA) and yeast strain Hf7C. The bait was a fusion of the GAL4 DNA-binding domain to either the 17-residue AF1 domain of EcR-B2, or the same fragment containing the mutation E9K. Binding was assayed as expression of β-galactosidase from a *UAS-lacZ* reporter.

### *In Vitro* Protein Binding

Proteins were synthesized *in vitro* using the TNT reticulocyte lysate kit (Promega, Madison, WI); template plasmids were described previously or were generated by a similar procedure [4]. Binding reactions (50 µl) contained 3 µl of each indicated translation mix in buffer A (20 mM NaH_2_PO_4_, 150 mM NaCl, pH 8.0) were incubated at 4° for 30 min. Then, 25 µl of a 50% slurry of Sepharose-protein A (Sigma) loaded with the indicated antibody was added and the incubation continued for 30 min. Beads were washed 3 times in buffer A and then boiled in SDS-PAGE sample buffer. The eluted proteins were separated by SDS-PAGE and radiolabeled proteins detected by autoradiography. For precipitation of full length EcRs, a mixture of the EcR-common region monoclonal antibodies, AG 10.2 and DDA 2.7 [6], was used with each at a 1∶10,000 dilution of an ascites fluid. For precipitation of GBT-B2-NS, we used a commercial antibody to the GAL4 DNA-binding domain (1∶1000, Santa Cruz Biotechnology, Santa Cruz, CA).

### Animals and Staging

*Drosophila melanogaster* were reared on standard cornmeal-yeast-agar media at 22–25° unless otherwise noted. Oregon-R was used as the wild-type strain. Larvae at the onset of metamorphosis were scored based on the blue color intensity observed in the gut of third instar larvae fed with cornmeal-yeast-agar food supplemented with 0.1% bromophenol blue. We collected blue gut larvae (18 hours before pupariation) and clear gut larvae (4 hours before pupariation) [32]. Prepupae and pupae were selected based on the criteria reported by Bainbridge and Bownes [33] at the following stages: white puparium (P1 stage; at puparium formation), buoyant prepupa (P4_i_ stage; 6.5–8 hours after puparium formation), and moving bubble prepupa (P4_ii_ stage; 12–13.5 hours after puparium formation).

The *ETH-GeneSwitch* (*ETH-GSW*) line was a kind gift from Michael Adams (University of California, Riverside) and Yoonseung Park (Kansas State University). It expresses a conditional, RU486-dependent GAL4 protein chimera [20] under the control of the 382 bp *ETH* promoter region [19], [21]. First instar larvae carrying *ETH-GSW* and selected UAS constructs were transferred after hatching to cornmeal-yeast-agar media supplemented with 500 mM RU486 [34]. In larvae, the expression of a reporter gene under *ETH-GSW*/RU486 control was restricted to just the Inka cells (Figure S1).

The CRC and EcR loss-of-function transgenes included *UAS-Crc-RNAi* (Vienna Drosophila RNAi Center (VDRC) line #2935, FBti0084038) [35], *UAS-EcR-B1-W650A* (FBti0026963), *UAS-EcR-B2-W650A*, *UAS-EcR-B1-F645A* (FBti0026961), and *UAS-EcR-B2-E9K*. The *UAS-EcR-B1* (FBti0023086), *UAS-EcR-B2* (FBti0023085), and *UAS-EcR-A* (FBti0023087) transgenes contain the three wild-type EcR isoforms [2].

### 20E Feeding

Freshly ecdysed *ETH-EGFP* (FBal0136020) third instar larvae were transferred on cornmeal-yeast-agar media supplemented with 0.08 mg/ml 20E [36] and collected 12 hours later for analysis of EGFP fluorescence in the Inka cells.

### Tissue Preparation and Image Analysis

Digoxigenin-labeled DNA probe preparation, whole-mount larval *in situ* hybridization, *ETH in situ* hybridization, anti-PETH immunostaining, and ETH-EGFP imaging was performed as described [19]. In larvae, the Inka cells are identified by the tracheal metameres (TMs) on which they are located, and the TMs are numbered 1 to 10, starting with the anterior end of the animal. To quantify the intensity of EGFP, immunostaining, and *in situ* hybridization signals, we measured the Intensity Index*Area = S*[(I–B)/B] where (S) is the surface area covered by the signal, (I) is the mean pixel intensity of the signal within this area, and (B) is the background signal intensity [19], [37]. This method takes into consideration the density of the signal distributed over the cell area, and it therefore normalizes for the angle at which the Inka cell is photographed and for heterogeneity in the spatial distribution of the signal. The measurements were taken using Adobe Photoshop (San Jose, CA, USA).

### Statistical Analysis

Statistical tests were performed using the NCSS 2001 software package (Kaysville, UT). Bonferroni corrections were performed to minimize type I errors in multiple pair-wise comparisons (Rice, 1989). We used parametric statistics because the data generally followed a normal distribution. All values are means ± s.e.m., except as indicated.

## Supporting Information

Figure S1 The *ETH-GeneSwitch* driver directed transgene expression specifically to the Inka cells. The image is a 2D confocal z-series projection of a larva expressing *UAS-mCD8::GFP* (FBti0012685) under the control of *ETH-GeneSwitch*. The larva was raised on food containing RU486. Expression of mCD8::GFP was limited to the Inka cells. The cells in tracheal metameres (TM) 1 and 4–9 on one side are labeled with arrows. The additional signal in the gut was due to yellowish autofluoresence. Bar = 200 µM.
(TIF)

Figure S2 EcR-B2-E9K rescued ETH protein expression and permitted ETH secretion. (A) In TM5, ETH protein expression in Inka cells expressing the dominant negative EcR-B1-F645A isoform was rescued by co-expression of EcR-B2-E9K but not wild-type EcR-B2. Means with the same lower case letters (TM5) were not significantly different (p>0.05). All larvae were fed RU486 and were dissected at ∼12 hr after ecdysis to the third instar. One-way ANOVAs (TM5, p = 0.004955; TM8, p = 0.125) were performed with Bonferroni (all-pairwise) multiple comparison post-hoc tests (n = 4–8). (B) In Inka cells expressing the dominant negative EcR-B1-F645A isoform, a reduction in ETH immunostaining consistent with ETH secretion was observed following rescue by EcR-B2 and EcR-B2-E9K. All animals were fed RU486 as larvae and were dissected either 9 hr after puparium formation (“pre-HE”) or 30 min after head eversion (“post-HE”). Head eversion (pupal ecdysis) occurs at approximately 12–13.5 hr after puparium formation [33]. Control animals had the same genotype as the EcR-B2 rescue animals, but were not fed RU486. One-way ANOVAs (TM5, p = 0.005087; TM8, p = 0.007636) were performed with Bonferroni (all-pairwise) multiple comparison post-hoc tests (n = 6). Note: Different developmental stages and confocal imaging settings were used for the experiments in panel (A) versus in (B), and the relative protein levels cannot be directly compared between these experiments.
(TIF)
