# Supplementary figures and images for: Cryptocephal, the Drosophila melanogaster ATF4, Is a Specific Coactivator for Ecdysone Receptor Isoform B2
Source: PLoS Genet. 2012 Aug 9;8(8):e1002883. doi: 10.1371/journal.pgen.1002883 (PMC3415445; doi:10.1371/journal.pgen.1002883)

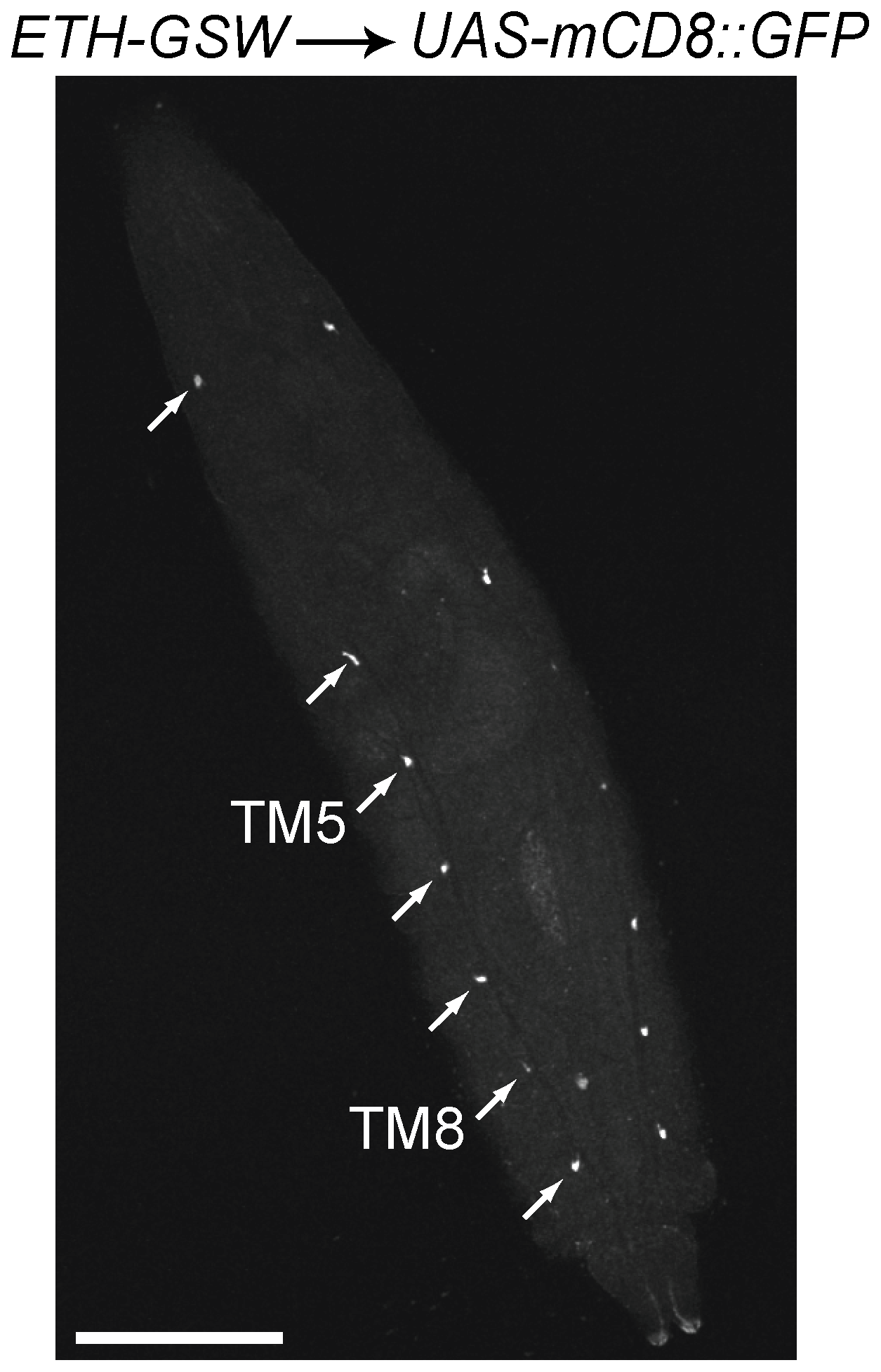

Supplement: Figure S1 — The ETH-GeneSwitch driver directed transgene expression specifically to the Inka cells. The image is a 2D confocal z-series projection of a larva expressing UAS-mCD8::GFP (FBti0012685) under the control of ETH-GeneSwitch. The larva was raised on food containing RU486. Expression of mCD8::GFP was limited to the Inka cells. The cells in tracheal metameres (TM) 1 and 4–9 on one side are labeled with arrows. The additional signal in the gut was due to yellowish autofluoresence. Bar = 200 µM. (TIF) [file pgen.1002883.s001.tif]

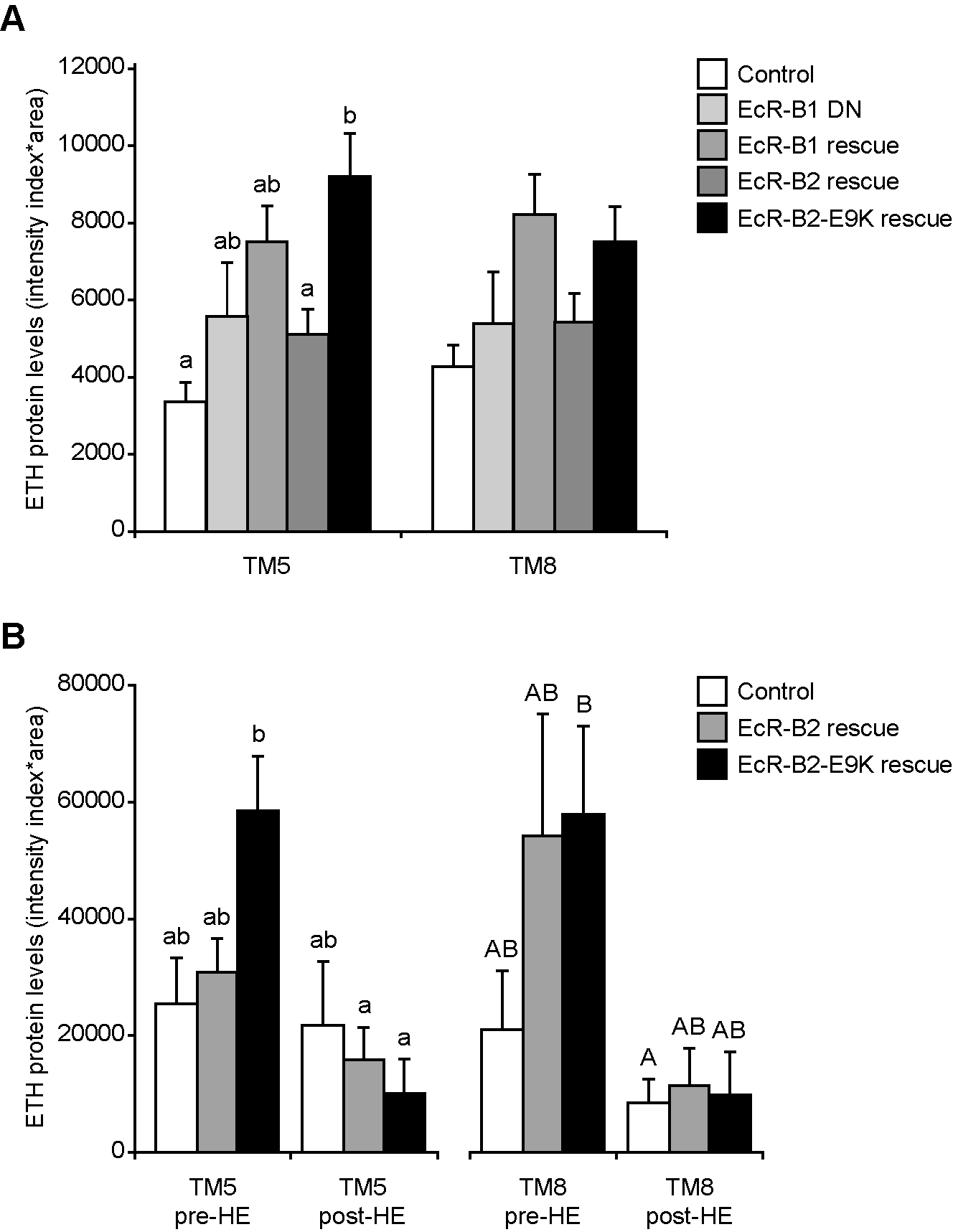

Supplement: Figure S2 — EcR-B2-E9K rescued ETH protein expression and permitted ETH secretion. (A) In TM5, ETH protein expression in Inka cells expressing the dominant negative EcR-B1-F645A isoform was rescued by co-expression of EcR-B2-E9K but not wild-type EcR-B2. Means with the same lower case letters (TM5) were not significantly different (p>0.05). All larvae were fed RU486 and were dissected at ∼12 hr after ecdysis to the third instar. One-way ANOVAs (TM5, p = 0.004955; TM8, p = 0.125) were performed with Bonferroni (all-pairwise) multiple comparison post-hoc tests (n = 4–8). (B) In Inka cells expressing the dominant negative EcR-B1-F645A isoform, a reduction in ETH immunostaining consistent with ETH secretion was observed following rescue by EcR-B2 and EcR-B2-E9K. All animals were fed RU486 as larvae and were dissected either 9 hr after puparium formation (“pre-HE”) or 30 min after head eversion (“post-HE”). Head eversion (pupal ecdysis) occurs at approximately 12–13.5 hr after puparium formation [33]. Control animals had the same genotype as the EcR-B2 rescue animals, but were not fed RU486. One-way ANOVAs (TM5, p = 0.005087; TM8, p = 0.007636) were performed with Bonferroni (all-pairwise) multiple comparison post-hoc tests (n = 6). Note: Different developmental stages and confocal imaging settings were used for the experiments in panel (A) versus in (B), and the relative protein levels cannot be directly compared between these experiments. (TIF) [file pgen.1002883.s002.tif]
